# Alterations of Regional Homogeneity and Functional Connectivity Following Short-Term Mindfulness Meditation in Healthy Volunteers

**DOI:** 10.3389/fnhum.2019.00376

**Published:** 2019-10-18

**Authors:** Qin Xiao, Xingrong Zhao, Guoli Bi, Lisha Wu, Hongjiang Zhang, Ruixiang Liu, Jingmei Zhong, Shaoyuan Wu, Yong Zeng, Liqian Cui, Yanmei Chen, Kunhua Wu, Zhuangfei Chen

**Affiliations:** ^1^Medical Faculty, Kunming University of Science and Technology, Kunming, China; ^2^Mental Health Institute, The Second Xiangya Hospital, Central South University, Changsha, China; ^3^Department of Psychiatry, The First Affiliated Hospital of Kunming Medical University, Kunming, China; ^4^Department of Magnetic Resonance Image, The First People’s Hospital of Yunnan Province, Kunming, China; ^5^Department of Clinical Psychology, The Second People’s Hospital of Yunnan Province, Kunming, China; ^6^Department of Clinical Psychology, The First People’s Hospital of Yunnan Province, Kunming, China; ^7^Department of Clinical Psychology, The Sixth Affiliated Hospital of Kunming Medical University, Yuxi, China; ^8^Department of Clinical Psychology, The First Affiliated Hospital of Sun Yat-sen University, Guangzhou, China

**Keywords:** emotion regulation, functional connectivity, mindfulness, regional homogeneity, default mode network, functional magnetic resonance imaging

## Abstract

Mindfulness is described as the non-judgmental awareness of experiences in the present moment. The sustained practice of mindfulness may also have beneficial effects on an individual’s well-being. For instance, mindfulness meditation is an effective approach for improving emotion regulation. Specifically, the early stage of mindfulness meditation training enhances emotional monitoring systems related to attention regulation and executive function. Reduced activity in the default mode network (DMN) would probably be observed corresponding to the attenuated mind wandering. In the present study, we hypothesized that alterations in functional activity in the frontal-parietal cortex and DMN may be induced by short-term mindfulness meditation. In this study, before and after 8 weeks of weekly Mindfulness-Based Stress Reduction (MBSR) training, healthy participants were evaluated using a mindfulness questionnaire and an affect schedule, as well as via resting-state functional magnetic resonance imaging. Sixteen right-handed non-meditators were enrolled. Another 16 demographically matched healthy adults without any meditation experience were recruited as controls. Pre- and post-MBSR assessments were compared. Increased regional homogeneity in the right superior parietal lobule and left postcentral gyrus (PoCG), as well as altered functional connectivity in PoCG-related networks, were observed post-MBSR. The mindfulness questionnaire scores also improved and negative affect was significantly decreased after MBSR. Together with reduced involvement of the posterior brain, our results suggest a tendency toward stronger involvement of the parietal cortex in mindfulness beginners. This study provides novel evidence regarding the optimization of emotional processing with short-term mindfulness meditation.

## Introduction

Mindfulness meditation has been demonstrated to improve emotion regulation (Teper et al., 2013). In the current clinical and research context, mindfulness meditation is defined as an attentive non-judgmental focus on and awareness of the present moment, particularly on internal sensations, feelings, and thoughts (Baer et al., 2006; Kong et al., 2016).

It is suggested that mindfulness acts via several underlying mechanisms (Hölzel et al., 2011), with attention and emotion regulation being two of the most regularly studied (Arch and Craske, 2006; Shapiro et al., 2006). Specifically, repeated mindfulness meditation training may bolster an individual’s self-regulation of attention and improve their emotional regulation (Lutz et al., 2015). Indeed, an improved underlying capacity for emotion regulation is precisely what mindfulness attempts to cultivate (Brown et al., 2013).

Self-regulation is a key process by which meditation fosters improved emotional functioning (Hölzel et al., 2011; Tang et al., 2015; Fox et al., 2016; Magalhaes et al., 2018). Recent neuroimaging studies focusing on attentional effects of meditation (especially, in the form of focused attention and open monitoring) revealed that improved emotion regulation was related to changes in neuronal activity in the brain (Guendelman et al., 2017). Furthermore, meditation induced changes to brain morphology and function in the dorsal anterior cingulate cortex, insula, dorsolateral prefrontal cortex, and the default mode network (DMN). Those regions are functionally related to general processes of self-regulation, attention assignment, enhanced body awareness, as well as introspection and enhanced metacognitive skills. Structural differences and reduced activity of the DMN might reflect less mind wandering and reduced chaining of thoughts in long-term meditators (Brewer et al., 2011b). Additionally, brain areas of the limbic system, including the amygdala, insula, ventral anterior cingulate cortex (vACC), and ventromedial prefrontal cortex (vmPFC), are also involved in emotional processing and the generation of affective responses. In fact, increased prefrontal activity and decreased activity of the limbic region have been shown during emotion regulation strategies such as cognitive reappraisal, implicit cognitive reappraisal, and attentional deployment (Magalhaes et al., 2018). Furthermore, a negative correlation between brain activations in prefrontal and right insula and mindfulness when viewing negative pictures suggests that less regulatory resources are needed in mindful individuals when facing emotional arousal (Lutz et al., 2014). Meditation training may also influence affective responses through amygdala reactivity, or connections among those regions (Herwig et al., 2018; Martelli et al., 2018; Tra et al., 2018).

By modifying emotion regulation abilities, mindfulness-related therapies, such as Mindfulness-Based Stress Reduction (MBSR) and Mindfulness-Based Cognitive Therapy (MBCT), reduce symptoms such as stress, anxiety, and depression, and ultimately benefit well-being (Baer, 2003; Brown and Ryan, 2003; Grossman et al., 2004). Significantly, MBSR has been shown to be effective in 8 weeks and induces similar structural and functional changes to the prefrontal cortex, the sensory cortices and insula, the hippocampus, and the cingulate cortex as long-term meditation practice (Gotink et al., 2016). It is not yet clear how short-term MBSR influences spontaneous neural activity (Chambers et al., 2009). While it has been proposed that the lateral prefrontal cortex and parietal cortex are involved at the very beginning of mindfulness meditation (Farb et al., 2007; Tang et al., 2009, 2012), the ACC is mainly involved when less effort is invested (Tang et al., 2009, 2012; Tang, 2011). Therefore, it is of great interest to clarify how the initial stages of mindfulness alter brain function.

Functional imaging studies indicate that mindfulness is associated with neural activity involving various brain regions, particularly those of the DMN (Brewer et al., 2011b). The DMN is involved in the internal channels of thoughts, mindfulness awareness, and interceptive awareness, and is critical for affective control (Shaurya et al., 2013). Mind wandering has been linked to increased activation in regions of the DMN (Weissman et al., 2006; Mason et al., 2007), such as in the mPFC, precuneus, and the posterior cingulate cortex (PCC). Negative emotions increase mind wandering (Jonathan et al., 2009), which in turn tends to feed negative mental states (Killingsworth and Gilbert, 2010). These negative loops are difficult to break, especially when they occur during pathological states such as depression, which has been linked to excessive DMN activity (Connolly et al., 2013). However, mindfulness meditation can provide a meta-function for an efficient brain/mind regulation, by flexibly allocating limited resources constrained by negative emotion and mind wandering. Even a few minutes of mindfulness practice can reduce mind wandering (Mrazek et al., 2012). Thus, we speculate that short-term practitioners of mindfulness have lower aggregate neural activity in the DMN.

On the basis of these previous findings, we hypothesized that (i) a short course of mindfulness practice would change neural activity in the brain regions related to attention and executive function, especially frontal-parietal regions, and (ii) a reduced recruitment of the DMN would be observed in practitioners. To gain insight about baseline neurobiological factors influencing sensory, cognitive, or behavioral processes associated with mindfulness (Mantini et al., 2007; Taylor et al., 2013; Wang et al., 2014; Kong et al., 2016), we measured spontaneous neural activity at rest using resting-state functional magnetic resonance imaging (rs-fMRI). To characterize the cohesiveness of resting activity in neighboring brain regions (focal connectivity), we adopted regional homogeneity (ReHo) as an analytic technique (Zang et al., 2004). ReHo supposes that voxels within a functional brain area are more temporally homogeneous when this area is involved in a specific condition. A larger ReHo value for a given voxel indicates a higher local synchronization of rs-fMRI signals among neighboring voxels. As a data-driven approach, ReHo can detect unpredicted hemodynamic responses that model-driven methods cannot. Thus, this method can help us understand the high complexity of the human brain (Zang et al., 2004). Additionally, functional connectivity was used to explore the long-range intrinsic organization of the brain function following MBSR (Han et al., 2017).

## Materials and Methods

### Participants

Sixteen right-handed non-meditators were enrolled as experimental participants and routinely attended MBSR practice for 8 weeks. An additional 16 healthy adults without any meditation experience were recruited as controls. Only individuals with no prior experience with meditation in any form (including mindfulness meditation and any other form of traditional practice like Shamatha, Satipatthana, yoga, Vipassana, Taiji) were enrolled. Demographically, the two groups were matched for age, sex, and years of education (*p* > 0.05) (Table 1).

**TABLE 1:** Demographic characteristics for all subjects.

	**MBSR group**	**CON group**
Number of participants	16	16
Gender (male/female)	8/8	8/8
Years of education	27.63 (1.25)	28.06 (1.60)
Educated (years)	17.00 (0.57)	15.17 (0.67)
Handedness (right/left)	16/0	16/0

*MBSR, mindfulness-based stress reduction; CON, control. Values are given as number or mean (standard error). No statistically significant differences between groups (*p* > 0.05).*

All individuals were recruited via poster advertisements in their local community. They were interviewed by two experienced psychiatrists who used the Structured Clinical Interview of the Diagnostic and Statistical Manual of Mental Disorders (DSM)-IV Axis I Disorders/Non-patient edition (SCID-I/NP) to exclude persons with a history of neuropsychiatric illness. All participants were Han Chinese and right-handed, as assessed via the Annett Handedness Scale (Annett, 1970). Persons with organic brain disorders, a history of alcohol or drug abuse, who were pregnant, or who had a history of, or present with severe physical illness were excluded.

The study was approved by the Ethical Committee of the Kunming University of Science and Technology (approval number: 2013JC003). All participants provided written informed consent before initiation of the experiment.

### Mindfulness Meditation Training

MBSR classes were conducted weekly for 2 h for a period of 8 weeks. These weekly meetings were divided into three segments: theoretical, practical, and debriefing. In the theoretical segment, MBSR and the underlying neuroscience were introduced to the participants. Next, in the practice segment, the participants performed a sitting meditation with simple physical and breathing exercises. Participants were instructed to focus their attention on their thoughts and feelings as they experienced them without ruminating on any of them.

Participants were also assigned daily homework assignments, which were composed of both formal and informal meditation activities. Formal activities required 30 min to complete each day and included body scanning, sitting meditation, floor yoga, mountain/lake meditation, or loving kindness meditation. Each week, the participants were also asked to complete a practice and information sheet, which was tailored to that particular week’s practice.

To integrate the participants’ learning and meditation practices into their daily lives, an informal practice that primarily included simple awareness activities (i.e., bringing mindful awareness to routine activities, pleasant/unpleasant events, or communication situations) was also undertaken. Additional techniques, such as the 1-min breathing space (Ward, 2014) and the Recognition, Acceptance, Investigation and Non-identification process (Brewer et al., 2011a), were incorporated into individuals’ informal practices to draw their attention toward their automatic responses and expand their awareness. At the end of each day, participants were instructed to spend approximately 5 min reflecting on their day, using that week’s informal practice sheet as a guide. On the other hand, the controls were asked to participate in a general health education course, with the same time setting as the MBSR.

### Self-Reported Measures

The Chinese versions of the Five Facet Mindfulness Questionnaire (FFMQ) (Deng et al., 2011) and the Positive and Negative Affect Schedule (PANAS) (Li and Yang, 2003) were applied both before and after the MBSR training to explore mindfulness and emotional states of all participants. The FFMQ assesses five facets of an individual’s general tendency to be mindful in daily life: observing, describing, acting with awareness, non-reactivity to one’s inner experience, and non-judgment of one’s inner experience. It consists of 39 items rated on a five-point Likert-type scale ranging from 1 (*never* or *very rarely true*) to 5 (*very often* or *always true*). The PANAS is a 20-item self-reported scale that measures positive and negative mood states in relation to the previous week. Both the negative and positive affect scales consisted of 10 corresponding adjectives that described either positive or negative emotions. Participants rated the degree to which they felt each emotion on a scale of 1 (*slightly* or *not at all*) to 5 (*a lot*).

### Functional Magnetic Resonance Imaging Data Acquisition and Preprocessing

Magnetic resonance imaging (MRI) data were acquired using a GE 3.0 T MR scanner (EXCLTE, General Electric, Milwaukee, WI, United States) using an eight-channel head coil. Participants were instructed to relax without engaging in any tasks and remain still with their eyes closed. All participants reported wakefulness throughout the duration of the scanning session. Functional data were collected using a contrast-gradient echo planar imaging (EPI) sequence with the following parameters: repetition time = 3000 ms, time to echo = 40 ms, time for inversion = 100 ms, flip angle = 90°, field of view = 240 mm × 240 mm, matrix size = 96 × 96, slice thickness = 4 mm, voxel size = 2.5 × 2.5 × 4 mm^3^, and 100 volumes in total (5 min and 30 s). No participants were found to have gross structural abnormalities upon visual inspection of their scans by two senior radiologists.

Preprocessing of imaging data was performed in MATLAB 7.6 (The MathWorks, Natick, MA, United States) using Statistical Parametric Mapping (SPM8^1^) and the Resting-State fMRI toolkit Data Processing Assistant (DPARSF v 2.2^2^). The first ten time-points from all functional images were removed to eliminate any non-equilibrium effects of magnetization. Slice timing and head motion corrections were also applied. All functional images were then normalized to the standard EPI template and spatially resampled to a voxel size of 3 × 3 × 3 mm^3^. The resulting images were detrended to remove the linear trend and then temporally filtered with a Chebyshev band-pass filter (0.01–0.08 Hz). Within the settings of DPRSF, nuisance factors including head motion parameters, white matter, global mean, and cerebrospinal fluid signals were regressed out.

### Regional Homogeneity Analysis

Kendall’s coefficient of concordance was used to measure the voxel-wise correlation between a single given voxel’s time series and its 26 neighbors. For standardization purposes, a Fisher’s r-to-z transformation was conducted on ReHo maps of the whole brain for all participants. All maps were then smoothed by applying a 6 mm full-width at half-maximum Gaussian kernel, as described previously (Yan and Zang, 2010). Voxel-wise comparisons of ReHo maps were performed to detect the inter- and intra- group differences. After fitting to the general linear model in SPM8, a two sample and a paired *t*-test were performed to identify the effects of meditation on ReHo between the MBSR and control (CON) group, as well as between the baseline and post-MBSR assessments, respectively.

### Region of Interest-Based Network Analyses

After ReHo comparisons, regions with significant ReHo abnormalities were selected as seeds for functional connectivity (FC) analysis. Seeds were defined as a sphere with a radius of 6 mm using the Montreal Neurological Institute and Hospital (MNI) stereotaxic coordinates. The mean time course of each seed was calculated by averaging the time series of all of the voxels within the seed. Voxel-wise correlation analysis was then performed between each of these selected time series and the rest. The FC maps were further transformed to z FC maps by Fisher’s r-to-z transformation to improve normality. The z FC maps in each condition were compared using paired *t*-tests. Additionally, eleven ROIs (defined as a sphere with a radius of 10 mm using the MNI coordinates, see Table 2) were ultimately selected for inclusion due to previous reports on their involvement in mindfulness meditation and the DMN (Allen et al., 2011; Doll et al., 2015). The averaged ReHo values for each ROI were extracted both before and after training for each participant. We tested for a main effect of MBSR training on ReHo across ROIs using a paired *t*-test in SPSS 13.0 (SPSS Inc., United States). Between-group ROI-based comparisons of ReHo maps were also performed between MBSR and CON groups using two sample *t*-tests.

**TABLE 2:** Regions of interest (ROIs) in DMN.

**Network**	**Region**	**Hemisphere**	**Abbreviation**	**MNI-coordinates**		
				***X***	***Y***	***Z***
aDMN	Anterior cingulate cortex	L	ACC.L	−6	53	1
	Superior frontal cortex(medial orbital)	L	ORBsupmed.L	−6	47	−8
	Anterior cingulate cortex	R	ACC.R	6	47	7
	Posterior cingulate cortex	L	PCC.L	0	−52	28
pvDMN	precuneus	R	PCUN.R	6	−61	28
	Posterior cingulate cortex	L	PCC.L	−6	−52	28
	Precuneus	L	PCUN.L	−6	−61	34
	Median cingulate cortex	R	MCC.R	12	−49	34
pdDMN	Precuneus	L	PCUN.L	−12	−67	34
	Precuneus	R	PCUN.R	9	−70	37
	Cuneus	L	CUN.L	−6	−76	34

*DMN, default mode network; aDMN, anterior DMN; pvDMN, posterior ventral DMN; pdDMN, posterior dorsal DMN.*

For all voxel-wise comparisons (ReHo analysis and seed-based FC analysis), results with a *p*-value of <0.05 at the voxel level or at the cluster level, after correcting for multiple comparisons and using the family-wise error (FWE) rate, were considered to be statistically significant. An additional extent threshold of 50 voxels was used to exclude any small clusters.

## Results

### Changes in Mindfulness Skills and Affective States

A paired *t*-test analysis of the total score and subscales (aside from the non-reactivity scores) of the FFMQ indicated a significant improvement in self-reported mindfulness after MBSR training (*p* < 0.05). The PANAS negative affect score also significantly decreased after training (*p* < 0.01) (Table 3).

**TABLE 3:** The five facet mindfulness questionnaire (FFMQ) and positive and negative affect schedule (PANAS) scores before and after meditation training.

	**Before training**	**After training**
**FFMQ**		
Observing	23.06 (1.54)	27.00 (1.27)^∗∗^
Describing	22.37 (1.09)	28.44 (1.19)^∗∗^
Acting with awareness	20.31 (1.15)	27.81 (1.11)^∗∗^
Non-reactivity	23.63 (1.32)	26.63 (1.22)
Non-judging	19.50 (1.09)	22.31 (1.31)^∗^
Total	108.876 (4.21)	132.19 (3.84)^∗∗^
**PANAS**		
positive affect	30.84 (0.86)	30.38 (0.95)
negative affect	16.63 (0.89)	12.88 (0.62)^∗∗^

*Values are given as group means (standard error). ^∗^*p* < 0.05, ^∗∗^*p* < 0.01.*

**FIGURE 1:**
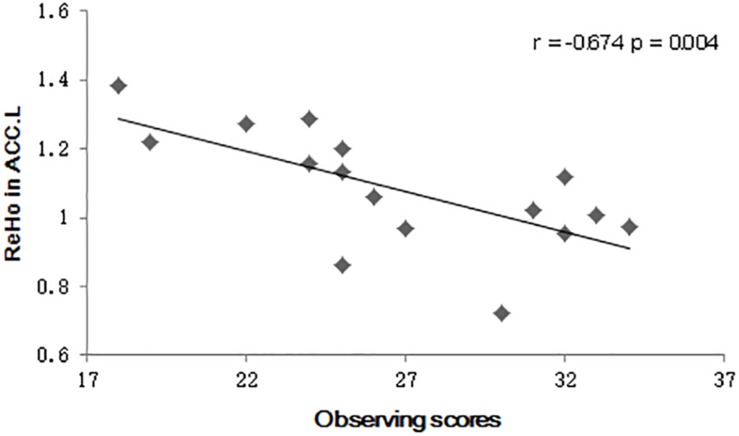
Scatter plot diagrams. The plots and fitted lines indicate the inverse correlations between the observed scores and the regional homogeneity (ReHo) values in the left anterior cingulate cortex (ACC) in the Mindfulness-Based Stress Reduction (MBSR) group.

As an exploratory step, the observing component score of the FFMQ was also found to negatively correlate with the mean ReHo value in the left ACC after MBSR training (*r* = −0.694, *p* = 0.003) (Figure 1). No correlation between the PANAS scores and the ReHo values was detected.

### Mindfulness and ReHo Differences

**FIGURE 2:**
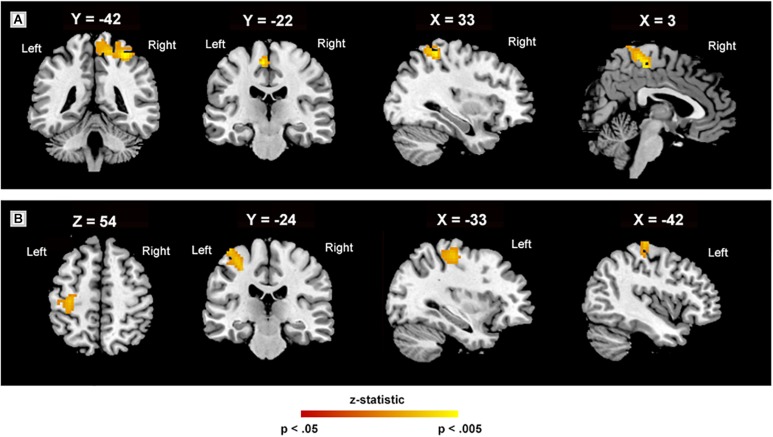
Regional homogeneity in various regions. Regional homogeneity peaks **(A)** in the right superior parietal lobule, extending into the left supplementary motor area and mid-cingulate gyrus (328 voxels); **(B)** in the left postcentral gyrus, extending into the precentral (126 voxels). The white labels indicate the coordinate of each slice in the Montreal Neurological Institute and Hospital (MNI) frame of reference (*x*, *y*, and *z*) (cluster level, threshold at *p* < 0.05, family-wise error corrected).

There were no significant differences in the CON group when ReHo was compared between the baseline and 8-week assessment (*p* > 0.05). In the MBSR group, comparison of the pre- and post-training images revealed increased ReHo values in the right superior parietal lobule (SPL.R) extending into the left supplementary motor area and mid-cingulate gyrus (MCC) (cluster size: 328 voxels, peak MNI coordinates: (*x* = 33, *y* = −42, *z* = 60, *t* = 6.70, *p* < 0.01), and the left postcentral gyrus (PoCG.L) extending into the precentral gyrus (cluster size: 126 voxels, peak MNI coordinates: (*x* = −33, *y* = −24, *z* = 54), *t* = 5.17, *p* = 0.01) after a cluster-level FWE correction (Figure 2). ReHo was not statistically different between the MBSR and CON group (*p* > 0.05).

**FIGURE 3:**
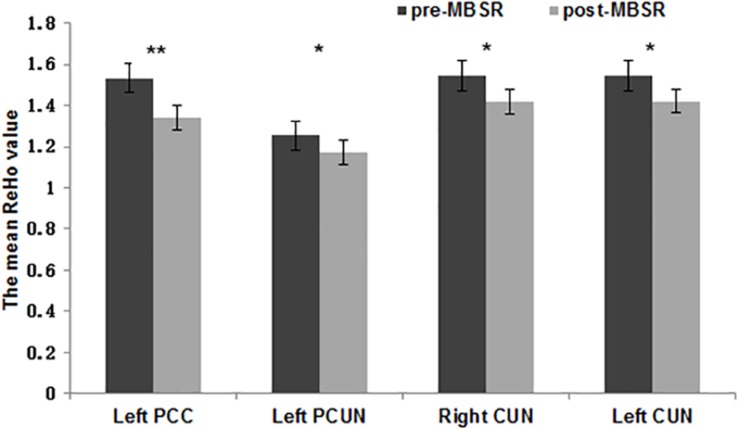
Regional homogeneity values of various regions of interest. The normalized mean regional homogeneity (ReHo) values of regions of interest (ROIs) in the left posterior cingulate cortex (x = –6, *y* = 52, *z* = 28), left precuneus (*x* = –12, *y* = –67, *z* = 34), right cuneus (*x* = 9, *y* = –70, *z* = 37), and left cuneus (*x* = –6, *y* = –76, *z* = 34). The error bars indicate the standard error. ^∗^*p* < 0.05, ^∗∗^*p* < 0.01.

To estimate the regional ReHo values in distinct areas, an ROI-level ReHo analysis was also conducted. Comparison of the mean ReHo values for each ROI in the DMN (Table 2) before and after MBSR revealed significantly decreased ReHo values in the left PCC (*t* = 3.09, *p* = 0.008), bilateral precuneus (*t* = 2.24, *p* = 0.04; *t* = 2.32, *p* = 0.04) in the pdDMN and left cuneus (*t* = 2.26, *p* = 0.04) (Figure 3). No significant difference was observed pre- and post-intervention in the CON group nor between the MBSR and CON groups both in baseline and after 8 weeks (*p* > 0.05).

### Pre- and Post-MBSR Comparison of Functional Connectivity

**FIGURE 4:**
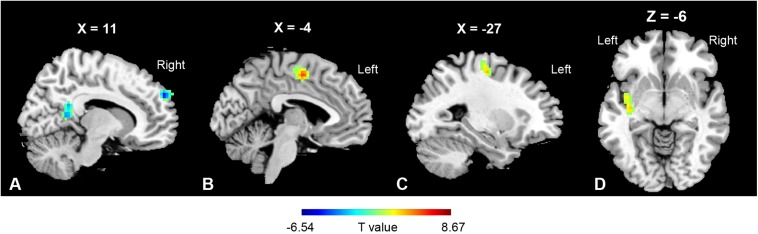
Regions of functional connectivity alterations linked to PoCG.L. PoCG.L related functional connectivity with a significant decrease was observed in **(A)** right precuneus (200 voxels) and right superior frontal medial gyrus (163 voxels), while that in the **(B)** mid-cingulate gyrus (126 voxels), **(C)** precentral gyrus (153 voxels), and **(D)** insula (103 voxels) in the left side was increased.

Based on the voxel-wise ReHo analysis, the SPL.R and PoCG.L were selected as seeds for network analysis. No significant differences were found between the MBSR and CON group both at baseline and after training, nor within the CON group following training (*p* > 0.05). In the MBSR group, however, the PoCG-related network showed both increased and decreased FC with the seed region. FC in the right precuneus (cluster size: 200 voxels, peak MNI coordinates: (*x* = 12, *y* = −51, *z* = 9, *t* = −5.57, *p* < 0.01) and right superior frontal medial gyrus (cluster size: 163 voxels, peak MNI coordinates: (*x* = 12, *y* = 57, *z* = 33, *t* = −6.54, *p* < 0.01) decreased, while the MCC (cluster size: 126 voxels, peak MNI coordinates: (*x* = −1, *y* = −6, *z* = 51, *t* = 8.67, *p* < 0.01), precentral gyrus (cluster size: 153 voxels, peak MNI coordinates: (*x* = −27, *y* = −15, *z* = −54, *t* = 5.75, *p* < 0.01) and insula (cluster size: 103 voxels, peak MNI coordinates: (*x* = −39, *y* = −3, *z* = −6, *t* = 5.10, *p* < 0.01) in the left side showed increased FC to PoCG, after FWE correction (Figure 4).

## Discussion

The goal of the present study was to investigate mindfulness-related functional brain changes in meditation-naïve participants. ReHo and ROI-based functional connectivity methods were used to explore intrinsic neural changes in meditation practitioners. Increased local synchrony in the bilateral parietal lobe was detected, as was reduced synchrony at the ROI-level (per ReHo analyses) in the PCC, precuneus, and cuneus, with meditation. A network-level analysis further revealed increased functional connectivity between the left PoCG.L and the MCC, insula and precentral gyrus of the left side in practitioners.

The observation that MBSR increased ReHo values in the bilateral parietal lobes is noteworthy. In our neuroimaging results, the largest cluster was observed in the right SPL. As a part of the attention and executive control networks, SPL is responsible for voluntary orienting of attention and coordinating of attention under competing situations (Corbetta and Shulman, 2002; Wadden et al., 2018). Mindfulness practice (e.g., breathing-awareness task) demands a high attentional state in which the practitioner is asked to orient his/her attention on breathing and related physical sensations such as temperature changes (Crivelli et al., 2019). Such focused attention in meditation practices fosters concentration, focusing, and self-regulation skills (Lutz et al., 2008; Lippelt et al., 2014; Hommel and Colzato, 2017). It has been shown that attention regulation is positively linked to emotion regulation (Wadlinger and Isaacowitz, 2011), and therefore the increased involvement of the superior parietal cortex may explain the MBSR group’s ability to selectively attend to their internal and external environment with awareness. A recent study similarly reported that the superior parietal cortex is causally involved in top-down allocation of attentional resources between external, ongoing stimuli and internal, prospective memory intentions; thus, it may lead to delayed intention (Cona et al., 2017). Furthermore, a recent multimodal neuroimaging study indicated that subregions of the SPL have different types of connectivity, reflecting different functions and interactions (Wang et al., 2015). The cluster we found in the right SPL may correspond to R1 (one of the anterior subregions), which has been associated with motion detection, observation, space, execution, shape detection, language orthography, sexuality, and working memory. Furthermore, SPL was found to regulate visuospatial attention and dominance of visuospatial attention (Wu et al., 2016). Meanwhile, engaging in mindfulness meditation training improves goal-directed visuospatial attention (Malinowski et al., 2017). Thus, it is of great importance to further elucidate the specific functions of SPL related to mindfulness.

Another cluster with increased ReHo value was located in the left PoCG. With the somatosensory cortex situated in it, the PoCG has a dominant role in processing sensory information from various parts of the body (Ruben et al., 2001; Eickhoff et al., 2010). In addition to processing somatosensory information, this brain area also modulates tactile attention (Burton et al., 1999), integrates somatosensory information with motor information, and processes pain (Timmermann et al., 2001). The somatosensory cortex also plays an important role in each stage of emotional processing, including identification of the emotional significance of a stimulus, the generation of empathy (Ilaria et al., 2007) and emotion (Adolphs et al., 2000; Damasio et al., 2000), as well as regulation of emotion (Satpute et al., 2015; Orenius et al., 2017).

Our result of increased ReHo values in parietal regions is consistent with data indicating that Hinduism-inspired meditation triggered a network of areas including the PoCG and the SPL (Tomasino et al., 2014). Parietal regions also support greater emotional stability in experienced meditators (Bruder et al., 1997; Broderick, 2005; Ochsner and Gross, 2008; Sheline et al., 2009). As outlined in the introduction, mindfulness improves the regulation of attention and emotion. In fact, an attention network composed of frontal-parietal regions, such as the SPL and pre-supplementary motor area, was found to be recruited by mindfulness meditation (Dickenson et al., 2013). Additionally, cognitive emotion regulation strategies may build upon more general information processing systems, such as those regulating working memory and cognitive control, to improve the functioning of these processes (Ochsner and Gross, 2008). Specifically, the SPL facilitates cognitive emotion regulation by manipulating and rearranging information in working memory stores (Koenigs et al., 2009). A previous study also found increased neural responses in attention-related parietal cortex (including SPL) when implementing attention regulation of negative self-beliefs (Goldin et al., 2013).

The intra-group analysis of ROI-based functional connectivity also revealed strengthened connectivity between PoCG and parietal as well as paralimbic regions in the left brain. In the study of Hinduism-inspired meditation, a left lateralized network was triggered. Brain areas reported to be activated in the literature overlapped with those of enhanced functional connectivity in our study, including the PoCG, the superior parietal lobe, and the mid-cingulate cortex (Tomasino et al., 2014). An increased FC was also detected between the insula and PoCG in the current study. Both MCC and insula are associated with mindfulness-related treatment (Shackman et al., 2011; Marchand, 2012; Zeidan et al., 2014) for the roles in the interoceptive processing (Craig, 2003; Vitaly et al., 2013). Besides, sensorimotor cortex, midcingulate, and insula belong to the same interoceptive network (Hayes et al., 2013). The decreased FC in the right precuneus and superior frontal medial gyrus may indicate a weakened association between attention (Nagahama et al., 1999) and somatosensory processing. However, it remains unclear how those areas work together in mindfulness-related interoceptive processing. Future studies may explore the underlying mechanism by implementing specific task-based experiments.

Taken together, our findings and prior work raise the possibility that parietal regions may subserve the improved processing of cognitive and emotional information observed with mindfulness.

At the ROI-level, the present study revealed decreased ReHo values in the PCC (precuneus, and cuneus; i.e., the posterior DMN) with mindfulness meditation. Our result is consistent with previous reports of reduced activation of DMN in experienced meditators (Brewer et al., 2011b).

The DMN encompasses a set of brain regions including the medial prefrontal cortex (mPFC) and the ACC, lateral parietal regions including the angular gyrus, and the PCC/precuneus. The DMN is normally deactivated during tasks requiring attention to external stimuli and activated during unconstrained thought, introspection, and self-related processing (Alex et al., 2012). The functions of the individual brain regions of the DMN are crucial to human mental activity (Long et al., 2008). Unsurprisingly, mindfulness, as a special form of mental activity, was also associated with functional changes in DMN-related areas.

Reduced DMN activity is normally observed during cognitively demanding tasks. The more attentional resources are needed, the greater the activation decrease (McKiernan et al., 2003). As the brain’s dark energy, the DMN contributes to greater memory task performance when deactivated (Kim et al., 2010). Poorer task performance was associated with reduced deactivation of this background activity, which may reflect mind wandering interfering with attention to external stimuli (Weissman et al., 2006; Tom et al., 2008). Mind wandering and negative emotion could mutually reinforce each other (Jonathan et al., 2009; Killingsworth and Gilbert, 2010), and could form a vicious circle in certain psychopathological states. Mindfulness meditation was associated with less mind wandering (Mrazek et al., 2012), as well as decreased activation of the DMN (Brewer et al., 2011b). Our results replicate these prior findings of functional attenuation of DMN following meditation. Though no direct measurement of mind wandering was conducted in the current study, participants reported that they were less absent-minded and found it much easier to concentrate following mindfulness training. Objective measurement of mind wandering should be addressed in future studies with a strict experimental design. It has been postulated that enhanced regulation of mind wandering is an important stage in early mindfulness training that may lead to enhanced cognitive regulation and metacognition (Kerr et al., 2013). Though accumulating evidence of the benefits of mindfulness training may be erroneously interpreted as evidence that mind wandering has no benefits. However, we must also focus on the potential benefits of mind wandering, such as planning of the future and reflecting on past experience. We should examine how to achieve a balance between the pros and cons of mind wandering in the practice of mindfulness (Mrazek et al., 2012).

The PCC is associated with mind wandering; it is activated during “distraction” and deactivated during “concentration” (Brewer et al., 2013) in meditators. It is implicated in self-referential processing (Whitfield-Gabrieli et al., 2011), including past and future thinking (Andrews-Hanna et al., 2010). The PCC is also associated with various emotion and cognitive processes, including emotional processing, self-referential distortions in depression, anxiety, drug craving, and cognitive distortions in chronic pain (Brewer and Garrison, 2014). The PCC was deactivated when individuals focused on a particular task without involving self-referential evaluation (Brewer et al., 2013), which fits perfectly with the requirements of mindfulness. Similar PCC deactivation was found when individuals practiced paying attention in a non-evaluative manner. The key difference between paying attention and paying attention in a non-evaluative manner is the amount of self-referential evaluation (Brewer et al., 2013).

In a recent study in meditation-naïve subjects, the precuneus was found with increased cortical thickness and decreased amplitude of low-frequency, which correlated with the reduction of depression scores (Yang et al., 2019). Our result of decreased involvement of precuneus in the MBSR group, indicated by the reduced ReHo value, was in line with this previous report. Many fMRI studies have reliably reproduced the DMN by investigating the correlations in fMRI signals recorded from the precuneus (Peng et al., 2015). The anterior precuneus is involved in higher-order body image and attentional shifting (Cavanna and Trimble, 2006), which are consistent with the practices and goals of many meditations. In a meta-analysis on the effects of meditation, structural heterogeneities in the precuneus were found in meditation practitioners (Fox et al., 2014). Furthermore, the structural connectivity between the precuneus and other areas of the attentional network such as the superior parietal lobe, may be mediated by changes in white matter after brief meditation training (Fox et al., 2014). The precuneus is crucial in the integration of mental processing with cognitive control processes such as visuo-spatial imagery, episodic memory retrieval and self-directed operations (Cavanna and Trimble, 2006; Fox et al., 2014; Yang et al., 2019). In the precuneus, self-referential processing is associated with increased functional activity, which decreases during mindful self-awareness. To be exact, this region may be linked to present moment-centered, body-oriented awareness cultivated in many meditation traditions, particularly Vipassana (i.e., insight meditation) (Fox et al., 2014). Functional differences of the precuneus in the MBSR group of this current study may be a result of its involvement in higher-order integration of interoceptive and exteroceptive attention at an advanced level. This process is potentially mediated by self-processing and evaluation. As advocated in many meditation traditions, a greater present-centered awareness is finally achieved by transforming the view of the self (Fox et al., 2014). The precuneus has consistently been reported to be dysregulated in depression (Li et al., 2018). The role it plays in emotion regulation should be further studied.

A reduced ReHo value was also detected in the cuneus, which belongs to the basic visual processing regions. The role of the cuneus in the cognitive domain has been rarely considered until recently. At first, the cuneus was reported to be associated with the cognitive ability of insight (Tordesillasgutierrez et al., 2018). Moreover, it has been involved in mindfulness related structural and functional change. One study found that mindfulness-based intervention in Parkinson’s disease increased gray matter volume in cuneus (Pickut et al., 2013). Another study found larger gyrification within the right cuneus in meditators. A recent study reported that individuals with borderline personality disorder showed higher deactivation in a cluster extending bilaterally from the calcarine to the cuneus and superior occipital gyri after dialectical behavioral therapy-which encompasses mindfulness as its core skill (Carmona i Farrés et al., 2019). Future studies should focus on the relationship between occipital regions and mindfulness practice both during a cognitive task and at resting state.

Enhanced mindfulness ability was observed in MBSR practitioners in our study. Our results concur with previous studies with similar findings measured using FFMQ (Kearney et al., 2011; Soler et al., 2018). Moreover, the observing component score of the FFMQ was negatively correlated with the mean ReHo value in the left ACC after MBSR training in our study. Research indicates that the ACC is involved in both cognitive control and emotional regulation, especially in the regulation and inhibition of emotion responses (Tang et al., 2015). Since we failed to find functional alteration in the ACC, this explanation should be treated cautiously.

The fMRI signal measures a relative hemodynamic change. Activation of a certain area corresponds to increased hemodynamic response (Cramer et al., 1999; Bernard et al., 2002) and vice versa (Allison et al., 2000; Hamzei et al., 2002). In ReHo analysis, Kendall’s coefficient concordance (KCC) was used to measure the similarity of the time series of a given voxel to those of its nearest neighbors in a voxel-wise way. Though the character of the hemodynamic response needs to be further investigated, it is generally accepted that a changed KCC in functional studies implies a changed hemodynamic response. Thus, the variation of ReHo value related to mindfulness in the current study was equivalent to that reported in the form of a change in activation, though no direct study evaluated the change of ReHo value in the DMN until recently. The reduction of ReHo value in the current study implies reduced involvement of the target brain regions in the resting state among mindfulness practitioners.

In the current study, there were significant differences in spontaneous brain activity and functional connectivity between the pre- and post-MBSR comparison. However, no alteration in the brain function was detected in the intergroup comparison between the MBSR and the CON group. The intra-group variability was more notable than the intergroup one. The variability might have resulted from both the small sample size and the statistical method. The two-sample *t*-test and paired *t*-test were separately used for comparison of inter- and intra-groups. The statistical efficiency of the latter would be much greater than that of the former (Rowe, 2015). The difference in statistical power would be more evident due to the small sample size. Therefore, we postulated that the sample size was not sufficient to detect the difference between the groups, but would be more sensitive to intra-group difference due to the self-control design. While the paired *t*-test, which, in fact is a one-sample *t*-test (Xu et al., 2017) that may increase the sensitivity, also raises the possibility of false positives. Therefore, further research is needed with a larger sample size, to explore MBSR-related brain change.

While the results of the present study offer some profound and important conclusions, there are also some limitations that must be taken into consideration. First, our sample size was moderately small, which limited our ability to detect small differences between conditions and inflated our chances of revealing positive findings, even when a correction for multiple-tests was used. As to the technical parameters for images, only 100 volumes were collected for each scan, which could be limited in reflecting the intrinsic state of the brain. Additionally, this study was limited by the lack of an active comparison or clinical intervention group, which would have provided a basis for making stronger inferences regarding how MBSR modifies the behavioral and neural bases of different emotion regulation strategies. Further studies should employ larger participant populations and clinically oriented observations with a focus on emotional pathways. Future work should also include additional assessment modalities, such as cognitive batteries and electrophysiology, in order to deepen our understanding of the mechanisms that underpin the effects of mindfulness on emotion regulation.

## Conclusion

The present study illustrated that individuals who undergo mindfulness training exhibit significant differences in functional connectivity between regions of the DMN. Short-term mindfulness training was also associated with strengthened mindfulness abilities. These results shed light on the preferential role of the parietal cortex and related associated areas in mindfulness beginners, in particular. Considering the acceptability and cost of short-term mindfulness training, the current findings indicate the potential value of mindfulness training on clinical and public health in emotion regulation.

## Data Availability Statement

The datasets generated for this study are available on request to the corresponding author.

## Ethics Statement

This study was carried out in accordance with the recommendations of the guidelines for scientific research on human subjects of the Ethics Committee of the Kunming University of Science and Technology with written informed consent from all subjects. All subjects gave written informed consent in accordance with the Declaration of Helsinki. The protocol was approved by the Ethics Committee of the Kunming University of Science and Technology (the ethical approval number: 2013JC003).

## Author Contributions

QX and XZ: data collection, data processing, data analysis, statistical analysis, original manuscript drafting, and manuscript editing. GB and HZ: data processing and data analysis. LW, RL, and YC: data collection, data processing, and data analysis. JZ, SW, YZ, and LC: project conception and manuscript revision. ZC and KW: project conception, research design, and manuscript revision.

## Conflict of Interest

The authors declare that the research was conducted in the absence of any commercial or financial relationships that could be construed as a potential conflict of interest.

## Footnotes

1 http://www.fil.ion.ucl.ac.uk/spm/

2 http://rfmri.org/DPARSF
